# Endoparasite Infections in Pet and Zoo Birds in Italy

**DOI:** 10.1100/2012/253127

**Published:** 2012-03-12

**Authors:** Roberto Papini, Martine Girivetto, Marianna Marangi, Francesca Mancianti, Annunziata Giangaspero

**Affiliations:** ^1^Dipartimento di Patologia Animale, Profilassi e Igiene degli Alimenti, University of Pisa, Viale delle Piagge 2, 56124 Pisa, Italy; ^2^Dipartimento PrIME, University of Foggia, Via Napoli 25, 71100 Foggia, Italy

## Abstract

Faecal samples were individually collected from pet (*n* = 63) and zoo (*n* = 83) birds representing 14 orders and 63 species. All the samples were examined by faecal flotation technique. In a subgroup of samples (*n* = 75), molecular assays were also used to detect *Cryptosporidium* oocysts and *Giardia duodenalis* cysts. Overall, 35.6% of the birds harboured parasites (42.2% of zoo birds and 27% of pet birds), including Strongyles-Capillarids (8.9%), *Ascaridia* (6.8%), Strongyles (5.5%), *G. duodenalis* Assemblage A (5.3%), Coccidia (4.1%), *Cryptosporidium* (4%), *Porrocaecum* (2.7%), *Porrocaecum*-Capillarids (2%), and *Syngamus*-Capillarids (0.7%). The zoonotic *G. duodenalis* Assemblage A and *Cryptosporidium* were exclusively found in Psittaciformes, with prevalences of 10.3% and 7.7% within this bird group. Zoo birds were more likely to harbor mixed infections (OR = 14.81) and symptomatic birds to be parasitized (OR = 4.72). Clinicians should be aware of the public health implications posed by zoonotic *G. duodenalis* Assemblages and *Cryptosporidium* species in captive birds.

## 1. Introduction

Birds are an integral part of virtually every ecosystem and it is not surprising that they are commonly found in households and zoos all over the world. Birds can be parasitized by a wide variety of endoparasites, that is, nematodes, trematodes, cestodes, acanthocephalans, and protozoa [1–3]. Although parasites usually cause little or no distress to healthy individuals in the wild, parasitic infections are among the most common sanitary problems affecting captive birds, especially in high-density populations [4]. Due to an increased risk of exposure, parasites can lead to serious problems or even to death in birds recently brought into captivity, kept for prolonged periods in confined housings, and stressed by injuries, illnesses, or adaptation to new environments [5–7]. As it is important to identify and control parasite species capable of producing diseases in captive birds, there is a clear need for parasitological studies on avian species. However, although there is a large body of literature on avian medicine including parasitic diseases [1–3], little has been documented about the epidemiology of parasites in pet and zoo birds. According to the literature, there is only one survey in pet birds where the prevalence of each intestinal parasite population was examined in parallel [8]. Some published studies included case reports [9–11] or surveys on a single parasitic agent [12, 13], while others examined intestinal parasites in a limited range of zoo species [14–16]. Only a few coprological surveys were carried out in a wide range of avian species displayed at zoo settings [17–20].

Among all parasites of birds, a special attention should be given to *Cryptosporidium* species due to their possible involvement in public health [21, 22]. So far, 3 species (*Cryptosporidium meleagridis*, *Cryptosporidium baylei*, and *Cryptosporidium galli*) and 10 genotypes have been considered as possible agents of avian cryptosporidiosis [21]. Each of them can infect several avian species, but they differ in their host range and infection sites. Reported prevalences for cryptosporidiosis in bird populations show a great variability, ranging from 0% in zoo birds [23] to 49% in wild ducks [24]. Human cryptosporidiosis is mainly related to anthroponotic cycle with *Cryptosporidium hominis *and to zoonotic cycle with *Cryptosporidium parvum *from cattle but also, though less frequently, with other species including *C. meleagridis *and *C. baylei* [22].

Two species of *Giardia* (*Giardia ardeae* and *Giardia psittaci*) are recognized as etiologic agents of avian giardiasis worldwide [25–30], while *Giardia duodenalis* is the only species, within the *Giardia* genus, that is responsible for infection of humans and other mammals [31]. Currently eight distinct Assemblages or genotypes (A–H) have been identified within this species: Assemblages C to H appear to be restricted to animal hosts, while Assemblages A and B have been detected both in humans and several animal species [31].

Interestingly, the most zoonotic *Cryptosporidium *species (*Cryptosporidium parvum*) and the zoonotic Assemblages of *Giardia duodenalis *(A and B) were found in faeces of various avian species [32–35], which are more likely to serve as mechanical vectors of cysts and oocysts. Although pet and zoo birds share the same environment with humans (owners, pet shop workers, zoo staff, and visitors), there is little information about their possible role as source of environmental contamination with zoonotic species of *Cryptosporidium *and/or zoonotic genotypes of *G. duodenalis*. Some surveys reported *Giardia* cysts and/or *Cryptosporidium *oocysts in faeces from pet or zoo birds, but the species and genotypes of isolates remained unknown since the identification relied on microscopic techniques [8, 12, 23]. To the best of our knowledge, only one study has documented the prevalence of *G. duodenalis *in zoo birds by a PCR-based diagnostic method [33]. Similarly, there are only two studies that have documented the prevalence of *Cryptosporidium* in zoo birds with molecular identification of the isolates [33, 35] and one in exotic birds commercialized as pets [36]. 

In order to give further insights, the present study was undertaken to gain epidemiological data on parasites detectable by conventional stool examination in a population of pet and zoo birds. In addition, the occurrence of *Cryptosporidium* species and* G. duodenalis *genotypes was molecularly investigated due to their potential zoonotic implications.

## 2. Materials and Methods

### 2.1. Animals and Specimen Collection

Between February and May 2011, freshly voided faecal samples were collected from 63 pet birds and 83 zoo birds representing 14 orders and 63 species. Orders, scientific names, and common names of pet and zoo birds sampled are listed in Tables 1 and 2, respectively. Pet birds were kept in households or were for sale in pet stores. Each species was housed separately in cages or aviaries depending on their size. Zoo birds were living in the zoological garden named “Giardino Zoologico Città di Pistoia”, located about 3 km from Pistoia (Tuscany, Central Italy). This is one of the largest zoological gardens in Italy and covers a hilly area of about 7 ha where exotic as well as autochthonous species are found. Closely related species of Anseriformes, Ciconiiformes, and Pelecaniformes were housed together in open pond areas, according to their zoological order. Peafowls were free-roaming, while all the remaining birds were housed separately in aviaries according to species. At the time of sampling, the large majority (*n* = 136) of birds did not show any clinical sign, while 10 animals were symptomatic. Faeces were collected off the ground by utilizing sterile polystyrene spatulas immediately after visually observing a single bird defecates. Mostly in cases of small- (up to 15 cm in length) and medium- (15–40 cm in length) sized pet birds as well as zoo birds kept separately from other species, multiple droppings were pooled from a single animal to collect an adequate amount of faeces (at least 2 grams) for parasitological examination. A new sterile spatula was used for each animal to avoid cross-contamination. Individual samples were labelled with bird species, stored in insulated clean polythene bags, and then put in a cooler bag before being transported to the laboratory.

### 2.2. Laboratory Procedures

Immediately upon arrival, each sample was preserved at +4°C before processing and then examined by a routine faecal flotation method within 12 hours of collection. Briefly, a commercial sodium nitrate solution with specific gravity of 1.2 (Coprosol, CandioliFarmaceutici spa, Beinasco, TO, Italy) was used. Slides were microscopically screened at 100x and 400x magnification. Parasites were identified by their morphometric characteristics. The presence of trematode eggs cannot be detected by using the present flotation solution and thus was not investigated. Due to inadequate amounts of faeces, aliquots of only 75 samples were frozen and stored at −20°C pending the molecular assay for the detection of *Cryptosporidium *and *Giardia* species/genotypes, that is, 52 samples from zoo birds and 23 from pet birds as shown in Tables 1 and 2.

### 2.3. Molecular Investigation

#### 2.3.1. DNA Isolation

DNA was isolated from individual faecal samples collected from pet and zoo birds. Each faecal sample was broken up in distilled water, and the oocysts were concentrated. In brief, 3 ml of faecal suspension were layered on 2.5 ml of 1 M sucrose (specific gravity 1.11) in a 75 × 12 mm plastic tube and centrifuged at 400 g for 15 min at room temperature. The water-sucrose interface was carefully removed with a Pasteur pipette, washed in 4 ml of normal saline, and centrifuged at 600 g for 10 min. The resulting sediment was resuspended in 200 *μ*l of saline solution and subjected to three freeze/thaw cycles (liquid nitrogen 5 min, 95°C 5 min). DNA was extracted using QIAMP DNA Mini Stool Kit (QiagenGmbh, Germany) according to the manufacturer's instructions. The extracted DNA was eluted in 50 *μ*l of distilled water, and all the samples were stored at −20°C until the molecular analyses were performed.

#### 2.3.2. *Cryptosporidium* and *Giardia * Molecular Detection

All the DNA extracts were subjected to a diagnostic two-step seminested PCR assay capable of amplifying a 360 bp *Cryptosporidium *oocyst wall protein (COWP) fragment for *Cryptosporidium* species and genotypes. The primer pair for the first step was CRY15D (5′-GTA GAT AAT GGA AGR GAY TGT G-3′) and CRY9D (5′-GGA CKG AAA TRC AGG CAT TAT CYT G-3′), and primers for the second step were CRYINT2D (5′-TTT GTT GAA GAR GGA AAT AGA TGT G-3), with CRY9D. Both PCR steps were carried out in a total of 50 *μ*l, containing 10 *μ*l of genomic DNA (first step) or 5 *μ*l of a 1/40 dilution (determined to be optimal) of each CR15D-CRY9D amplicon (second step), 100 p mol of each primer and 25 *μ*l of Ready Mix RED Taq (Sigma, St. Louis, MO). Both amplification rounds consisted of an initial step of 12 min at 94°C, followed by 40 cycles, each of 50 s at 95°C, 40 s at 50°C, and 50 s at 72°C, with a final step of 7 min at 72°C.

All the DNA extracts were also subjected, in duplicate, to PCR assay capable of amplifying a partial *β*-giardin gene sequence of about 171 bp from *G. duodenalis*. Consensus primers were GGL (5′-AAGTGCGTCAACGAGCAGCT-3′) and GGR (5′-TTAGTGCTTTGTGACCATCGA-3′). Each PCR mixture of 25 *μ*l reaction mix contained 5 *μ*l of sample DNA, 12.5 *μ*l Ready Mix RED Taq (Sigma, St. Louis, MO), and 0.5 mM concentrations of each primer. The thermal cycling protocol was as follows: initial denaturation at 95°C for 4 min, followed by amplification for 40 cycles of 60 s at 95°C, 60 s at 61°C, and 60 s at 72°C, and 72°C for 7 min.

Samples of DNA extracted from *G. duodenalis* (American Type Culture Collection, ATCC 30957) or from known *Cryptosporidium*-positive stool specimens of a previous study [37] and samples with distilled water instead of template were included in PCR reactions as positive and negative controls. The resulting PCR products were electrophoresed on a 2% agarose gel and visualized by UV. The successful PCR reactions were further purified using Ultrafree-DA columns (Millipore, Billerica, MA) and sequenced by ABI PRISM 3130. The sequences were aligned with each other by using the Clustal X application and compared with those of *Giardia duodenalis *and *Cryptosporidium *species registered in the GenBank database by using the nucleotide-nucleotide BLAST tool available online at the National Center for Biotechnology Information website.

### 2.4. Statistical Analysis

A positive bird was defined as any animal testing positive for at least one endoparasitic species. Prevalence values were calculated as number of positive animals/number of examined animals ×100 with the corresponding 95% confidence intervals (95% CI). Differences between groups were compared by the chi-square test. *P* values <0.025 were considered significant. Odds ratio (OR) and corresponding 95% CI values were also calculated as a measure of the risk. Statistical values determined as not significant are not presented.

## 3. Results

By microscopy, nematode eggs were detected in 26.7% (19.5–33.9%) of the birds, with 32.5% (22.4–42.6%) in zoo birds and 19% (9.3–28.7%) in pet birds. The occurrence of parasites showed some variability between the two avian groups, since *Ascaridia* and *Syngamus *were identified only in pet birds while *Porrocaecum *only in zoo birds. Unsporulated coccidia oocysts were found in 4.1% (0.9–7.3%) of the samples, showing prevalences of 6.3% (0.3–12.4%) and 2.4% (0–5.7%) in samples from pet and zoo birds, respectively. Monoparasitoses (i.e., strongylosis, ascaridiosis, or coccidiosis) were present in 19.2% (12.8–25.6%) of the animals, including 23.8% (13.3–34.3%) of pet birds and 15.7% (7.8–23.5%) of zoo birds. Polyparasitoses with two nematode infections (i.e., capillariasis associated to strongylosis, ascaridiosis, or syngamosis) occurred in 11.6% (6.4–16.8%) of the birds. These included 19.3% (10.8–27.8%) of zoo birds and 1.6% (0–4.7%) of pet birds, reaching a statistically significant difference (*χ* ^2^ = 10.89, *P* = 0.0010, OR = 14.81 [1.91–114.97]). Spurious parasites (i.e., oxyurid eggs) were recovered in a faecal sample from an Eurasian eagle-owl (*Bubo bubo*).

Out of the 75 faecal samples examined by molecular assay, 3 (4%) and 4 (5.3%) samples from Psittaciformes were found to be positive for *Cryptosporidium *and* G. duodenalis*, respectively. Three (5.7%) samples from zoo birds produced amplicons of the expected size for *Cryptosporidium*, but the sequencing failed probably because of the poor quality of the template. These samples were from a blue-fronted amazon (*Amazona aestiva*), an eastern rosella (*Piatycercus eximius*), and an alexandrine parakeet (*Psittacula eupatria*). The comparison of the DNA sequences of the *β* giardin gene with those of *Giardia* available in the GenBank database showed that *G. duodenalis* Assemblage A (i.e., homology rate of 100%; accession number X85958) was found in 3 (5.7%) zoo birds and 1 (4.3%) pet bird. These included another blue-fronted amazon, a blue-and-yellow macaw (*Ara ararauna*), a scarlet macaw (*Ara macao*), and a white-bellied parrot (*Pionites leucogaster*). Taking into account 39 psittacine faecal samples (23 from pet parrots and 16 from zoo parrots) examined by molecular assay, *Cryptosporidium *was found in 7.7% (0–16.1%) of the birds and, in particular, in 18.7% (0–37.9%) of zoo parrots. The zoonotic Assemblage A of *G. duodenalis *was identified in 10.3% (0.7–19.8%) of them, showing a prevalence of 18.7% (0–37.9%) in zoo parrots and 4.3% (0–12.7%) in pet parrots. 

Overall, combining results of microscopy with those of molecular biology techniques, 52 out of 146 (35.6%) birds were found to harbour parasites. Distribution of parasites and their associations in pet and zoo birds are shown in Tables 1 and 2, respectively. Total numbers of positive samples, prevalence values, and 95% CI are summarized in Table 3. Seven out of 10 birds with clinical signs were found to be parasitized, as shown in Table 4. Statistical analysis showed a significant difference (*χ* ^2^ = 5.535, *P* = 0.0186, OR = 4.72 [1.16–19.11]) in the total prevalence of parasites between symptomatic and asymptomatic birds (70% [41.6–98.4%] versus 33.1% [25.2–41%]).

## 4. Discussion

The present findings show that parasites can be very common in zoo and pet birds, since 42.2% and 27% were shown to be coprologically positive, respectively, with some of them harbouring potentially zoonotic protozoa. Previous studies found endoparasites in 11.1–51.9% of zoo birds in Turkey [20], from 48.1% to 71.4% in India [17, 18], 51.6% in Spain [19], and in 22.5% of pet birds in Japan [8]. All the parasites found have faecal-oral route of transmission. Thus, contaminated soil, food, and water play a key role as sources of parasite infection to birds under captivity conditions.

The most frequently encountered eggs were those of strongyles. These are small, fine worms that occur in the caeca (*Trichostrongylus*) and gizzard (*Amidostomum*, *Epomidiostomum*) but also in the respiratory tract (*Cyathostoma*, *Syngamus*) of birds. Heavy infections with caecal and gizzard strongyles can lead to serious disease mostly in red grouses [38] and waterfowl within the family Anatidae [39], respectively. The clinical signs are unspecific, including anaemia, appetite loss, diarrhoea, dullness of the plumage, emaciation, general weakness, malnutrition, and unthriftiness [38, 39]. In this survey, none of the subjects harbouring single or mixed infections with strongyles showed clinical signs. Respiratory strongyles are usually few in number and not pathogenic but can occasionally cause dyspnea, emaciation, and open mouth breathing, mostly in young- and smaller-sized birds [40]. Clinical signs were present in a peafowl showing mixed infection with *Syngamus *and capillarids (Table 4). Since signs of respiratory distress were not evident, we believe that clinical signs were mostly attributable to capillarid infection. 

Avian parasites that belong to the genera *Baruscapillaria*, *Capillaria*, *Echinocoleus*, *Eucoleus*, *Ornithocapillaria*, *Pterothominx*, and *Tridentocapillaria *are collectively referred to as capillarids [41]. They include species that infect the oral cavity, pharynx, oesophagus, crop, small intestine, or caecum. Intestinal infections are usually asymptomatic, but birds with heavy parasite burden may show clinical signs of anorexia, diarrhoea, emaciation, reduced water intake, ruffled feathers, and weakness [41]. Other species, generally considered more pathogenic, can produce significant tissue damage by burrowing into the mucosal lining of the mouth, oropharynx, oesophagus, and crop. They cause dehydratation, diphtheritic membranes extending from the oral cavity to the proventriculus, emaciation, necrosis, oedema, and severe inflammation [41]. In the present survey, all capillarid-infected birds (*n* = 17) had mixed infections, and three of them showed clinical signs (Table 4). Interestingly, the statistical analysis revealed that zoo birds were about fifteen times more likely to develop mixed nematode infections than their pet counterpart. This was mainly evident in the group of Anseriformes, where a number of closely related species were housed all together, and was probably due to both higher risk of environmental contamination with multiple parasitic agents in the zoo situation and low host species specificity of many bird nematodes [38, 39, 41]. 

Ascarid eggs encompassed typical eggs of both *Ascaridia *and *Porrocaecum*. Ascarids are the largest nematodes infecting birds and generally inhabit the small intestine [42, 43]. In small number, they are usually not pathogenic causing only occasional unthriftiness. However, they can produce overt clinical disease and even death if their number is sufficiently large to cause anaemia, severe inflammatory response, and starvation [42, 43]. None of the *Ascaridia*-infected subjects showed clinical signs, while *Porrocaecum* was found not only in asymptomatic birds but also in two symptomatic gray crowned cranes (*Balearica regulorum*) concurrently infected with capillarids (Table 4). Due to the mixed infection, it was difficult to determine the role each parasitosis played in the occurrence of symptoms.

Intestinal coccidia occurring in birds include species of the genera *Eimeria*, *Isospora*, *Tyzzeria*, and *Wenyonella* [44]. They can be distinguished by the characteristic morphology of their sporulated oocysts that differ mainly in number of sporocysts and sporozoites [45]. In this study, unsporulated oocysts were found in faecal samples from three canaries, a budgerigar, and an emu belonging to the orders Passeriformes, Psittaciformes, and Casuariiformes. *Eimeria* and *Isospora* infections can occur in Passeriformes and Psittaciformes [44, 45]. Unidentified coccidia have been reported in Casuariiformes [45]. Neither *Tyzzeria* nor *Wenyonella* is known to occur in avian species belonging to these orders [44]. Therefore, the genera *Eimeria *and *Isopora* were thought to be the most likely cause of coccidia infection in this survey. Clinical signs of intestinal coccidiosis include watery, mucoid, or bloody diarrhoea, decreased egg production, emaciation, lack of appetite, lethargy, incoordination, ruffled feathers, and weight loss [45]. In the present survey, 4 out of 5 coccidia-infected birds showed clinical signs (Table 4).

As for *Cryptosporidium*, the present prevalence in zoo birds (5.7%) is higher than 0% [23] and 1.1% [33] but lower than 10% [35] previously found in zoo bird collections in Japan, Poland, or Malaysia, respectively. None of the pet birds of our survey tested positive for *Cryptosporidium*. A prevalence of 6.8% of cryptosporidiosis was reported in exotic birds that were for sale as pets in Brazil [36]. This is the first report on the occurrence of *Cryptosporidium* in birds in Italy. In our study, isolates of *Cryptosporidium* could not be identified to species level; however, it cannot be excluded that zoo birds may harbor also *Cryptosporidium *species that are specific to hosts other than birds, including *C. parvum*.

In Italy, the zoonotic Assemblage A of *G. duodenalis *has been identified in humans, dogs, cats, cattle, sheep, buffaloes, fallow deer, and water samples, as reviewed by Giangaspero and coworkers [37]. The current study is the first to report the occurrence of *G. duodenalis *in birds in this country. The prevalence of *G. duodenalis *registered in this study in zoo birds (5.7%) is higher than 2.2% in captive birds reared at the Poznan Zoological Garden in Poland [33] and 1.7% of *Giardia* infection found at the Osaka Zoological Garden in Japan [23]. On the contrary, the prevalence of *G. duodenalis *in pet birds of this survey (4.3%) is much lower than 16.1% of *Giardia* infection detected in passerines and psittacines commonly kept as pets in Japan [8].

It is worth nothing that all the birds carrying *G. duodenalis *cysts (5.3%) and *Cryptosporidium* oocysts (4%) in their faeces belonged to the order Psittaciformes. Thus, parrots may play a role in disseminating zoonotic *G. duodenalis* (Assemblage A) cysts and zoonotic *Cryptosporidium *species (*C. meleagridis*, *C. baylei*, and *C. parvum*) oocysts [32–35]. Whether they acquired *G. duodenalis *cysts and *Cryptosporidium* oocysts through ingestion of contaminated soil, water or food remains unknown. *G. duodenalis *Assemblages A and B have been identified in water samples collected in a zoo setting in Malaysia [46]. Trophozoites of an avian isolate of *G. duodenalis* from a sulfur-crested cockatoo were experimentally infective to mammals [47, 48]. Zoonotic *Cryptosporidium* species other than *C. parvum* have been implicated in human cases of cryptosporidiosis, including species that are considered specific for birds [22]. Since birds may have the potential for being mechanical transporters of cysts and oocysts without being infected themselves, it is important to accurately identify the avian species serving as transport hosts for a better understanding of the diffusion routes of *G. duodenalis* and *Cryptosporidium* in the environment and thus, ultimately, for a better control of human infections. Therefore, due to the possible public health implications posed by birds that harbor zoonotic Assemblages of *G. duodenalis* and zoonotic *Cryptosporidium *species, it would be advisable that children, elderly, and immunocompromised individuals do not come into contact with carrier birds or with environments contaminated by them. As zoo workers are highly exposed to risk of infection with zoonotic agents [48], it would be also recommended that people taking care of birds follow hygienic measures such as wearing gloves for cleaning cages and washing hands thoroughly after all routine management procedures.

In this study, the large majority (*n* = 45) of positive birds did not present any clinical sign, probably as a result of low parasite burdens. This shows that captive birds are not frequently affected by overt clinical parasitism. However, a rate as high as 70% of symptomatic birds was found to be positive for intestinal parasites by coprological examination. Symptomatic birds were about five times more likely to be parasitized than asymptomatic ones. Thus, the monitoring, diagnosis, and treatment of parasitic infections should be a routine part of the health care of pet and zoo birds, mostly when clinical signs are present.

Eggs and oocysts from prey species (spurious parasites) are often found when performing faecal examinations of raptors and can be similar to those of avian parasites. In the present study, eggs of oxyurids, common mouse worms, were observed in a faecal sample from Eurasian eagle-owl (*B. bubo*). Therefore, the interpretation of results of coprological examinations in raptors must be undertaken with special care.

To conclude, the present survey provides further insights into the epidemiology of internal parasites in birds. Our findings show that identification of parasites and establishment of their prevalence may be of paramount importance both in pet and zoo birds, even if some agents are referred to as low pathogenic. Adequate knowledge concerning epizootiology, transmission, pathogenicity, diagnosis, treatment, and control of avian endoparasites as well as awareness of public health concerns posed by birds harbouring zoonotic *G. duodenalis* assemblages and/or *Cryptosporidium* are thus required to clinicians working in exotic pet practices or zoo settings.

## Figures and Tables

**Table 1:** Orders, scientific names, common names, numbers examined, positive numbers, and intestinal parasites found in pet birds.

Order	Scientific name	Common name	No. examined	No. positive	Parasites
Columbiformes	*Columba livia*	Pigeon	1	0	—

Galliformes	*Pavo cristatus*	Peafowl	1	1	*Syngamus*-Capillarids

Passeriformes	*Carduelis carduelis*	European Goldfinch	1	0	—
	*Serinus canaria*	Canary	5	3	Coccidia
	*Turdus merula*	Black bird	1	0	

Psittaciformes	*Agapornis fischeri*	Fischer's Lovebird	2	0	
	*Agapornis nigrigenis*	Black-cheeked Lovebird	7 (6*)	0	
	*Agapornis roseicollis*	Rosy-faced Lovebird	3*	0	
	*Agapornis personata*	Masked Lovebird	1	0	
	*Amazona aestiva*	Blue-fronted Amazon	3 (1*)	1	*Ascaridia*
	*Aratinga canicularis*	Orange-fronted Parakeet	1*	0	
	*Aratinga acuticaudata*	Blue-crowned Parakeet	1	0	
	*Aratinga jandaya*	Jandaya Conure	1	0	
	*Aratinga solstitialis*	Sun Conure	3 (1*)	0	
	*Bolborhynchus lineola*	Barred Parakeet	2*	1	*Ascaridia*
	*Cyanoliseus patagonus*	Burrowing Parakeet	1*	0	
	*Eos bornea*	Red Lory	1	1	Strongyles
	*Melopsittacus ondulatus*	Budgerigar	7	1	Coccidia
	*Myiopsitta monachus*	Monk Parakeet	2 (1*)	1	*Ascaridia*
	*Neopsephotus bourkii*	Bourke's Parrot	2	2	*Ascaridia*
	*Nymphicus hollandicus*	Cockatiel	1	0	
	*Pionites leucogaster*	White-bellied Parrot	4 (1*)	3	*Ascaridia* (2), *G. duodenalis* Ass^#^ A (1)
	*Pionites melanocephalus*	Black-headed Parrot	3 (2*)	2	*Ascaridia*
	*Platycercus eximius*	Eastern Rosella	1	0	
	*Poicephalus senegalensis*	Senegal Parrot	4 (3*)	1	*Ascaridia*
	*Psittacula krameri*	Rose-ringed Parakeet	2 (1*)	0	
	*Psittacus erithacus*	African Grey Parrot	2	0	
	*Trichoglossus haematodus*	Rainbow Lorikeet	1	0	

Total			63 (23*)	17	

*Number of birds examined for *Cryptosporidium *species and* G. duodenalis* genotypes; ^#^Assemblage.

**Table 2:** Orders, scientific names, common names, numbers examined, positive numbers, and intestinal parasites found in zoo birds.

Order	Scientific name	Common name	No. examined	No. positive	Parasites
Anseriformes	*Anas crecca*	Common Teal	1*	0	
	*Anser albifrons*	Greater White-fronted Goose	1*	1	Strongyles-Capillarids
	*Anser cygnoides*	Swan Goose	1*	1	Strongyles-Capillarids
	*Anser indicus*	Bar-headed Goose	1	1	Strongyles-Capillarids
	*Brantacanadensis*	Canada Goose	1*	1	Strongyles-Capillarids
	*Cereopsis novaehollandiae*	Cape Barren Goose	2 (1*)	2	Strongyles-Capillarids
	*Coscoroba coscoroba*	Coscoroba Swan	2 (1*)	2	Strongyles-Capillarids
	*Cygnus atratus*	Black Swan	1*	0	
	*Netta rufina*	Red-crested Pochard	1	0	

Casuariiformes	*Dromaius novaehollandiae*	Emu	2*	2	Coccidia

Ciconiiformes	*Egretta garzetta*	Little Egret	2 (1*)	1	Strongyles
	*Eudocimus ruber*	Scarlet Ibis	4 (2*)	2	Strongyles
	*Ciconia ciconia*	White Stork	4 (1*)	2	*Porrocaecum*
	*Nycticorax nycticorax*	Black-crowned Night-heron	2 (1*)	1	Strongyles
	*Threskiornis aethiopicus*	Sacred Ibis	3 (1*)	2	*Porrocaecum*

Coraciiformes	*Dacelo novaeguineae*	Laughing Kookaburra	2 (1*)	0	

Falconiformes	*Parabuteo unicinctus*	Harris's Hawk	1*	0	

Galliformes	*Lophura swinhoii*	Swinhoe's Pheasant	1*	0	
	*Pavo cristatus*	Peafowl	10 (7*)	5	Strongyles-Capillarids

Gruiformes	*Anthropoides virgo*	Demoiselle Crane	1*	1	*Porrocaecum*-Capillarids
	*Balearica regulorum*	Grey Crowned Crane	2	2	*Porrocaecum*-Capillarids

Passeriformes	*Corvus monedula*	Jackdaw	1*	1	Strongyles

Pelecaniformes	*Pelecanus onocrotalus*	Great White Pelican	3*	0	
	*Phalacrocorax carbo*	Great Cormorant	2	0	

Psittaciformes	*Amazona aestiva*	Blue-fronted Amazon	6 (5*)	2	*G. duodenalis* Ass^#^ A (1), *Cryptosporidium *sp (1)
	*Amazona ochrocephala*	Yellow-crowned Amazon	2	0	
	*Ara ararauna*	Blue-and-yellow Macaw	4*	1	*G. duodenalis* Ass A
	*Ara chloroptera*	Green-winked Macaw	2 (1*)	0	
	*Ara macao*	Scarlet Macaw	2 (1*)	2	Strongyles (1), *G. duodenalis* AssA (1)
	*Aratinga leucophthalmus*	White-eyed Conure	2 (1*)	0	
	*Nymphicus hollandicus*	Cockatiel	2 (1*)	0	
	*Platycercus eximius*	Eastern Rosella	1*	1	*Cryptosporidium *sp
	*Psittacula eupatria*	Alexandrine Parakeet	2*	1	*Cryptosporidium *sp

Rheiformes	*Rhea americana*	Greater Rhea	2*	0	

Strigiformes	*Bubo africanus*	Spotted Eagle-owl	1	1	Strongyles
	*Bubo bengalensis*	Indian Eagle-owl	1*	0	
	*Bubo bubo*	Eurasian Eagle-owl	3 (2*)	1^§^	Oxyurids^§^
	*Tyto alba*	Barn Owl	1*	0	

Struthioniformes	*Struthio camelus*	Ostrich	1	0	

Total			83 (52*)	35	

*Number of birds examined for *Cryptosporidium* species and* G. duodenalis* genotypes; ^#^Assemblage; ^§^Spurious parasites were not included in the total count.

**Table 3:** Number of positive samples, prevalence, and 95% confidence intervals (95% CI) of intestinal parasites in pet and zoo birds.

Parasites	Pet birds (*n* = 63)			Zoo birds (*n* = 83)			Total (*n* = 146)		
	No. positive	Prevalence	95% CI	No. positive	Prevalence	95% CI	No. positive	Prevalence	95% CI
Strongyles-Capillarids	0	—	—	13	15.7%	7.8–23.5%	13	8.9%	4.3–13.6%
*Ascaridia*	10	15.9%	6.8–24.9%	0	—	—	10	6.8%	2.7–10.9%
Strongyles	1	1.6%	0–4.7%	7	8.4%	2.5–14.4%	8	5.5%	1.8–9.2%
*G. duodenalis *Ass* A^#^	1	4.3%	0–12.7%	3	5.7%	0–12.1%	4	5.3%	0.2–5.4%
Coccidia	4	6.3%	0.3–12.4%	2	2.4%	0–5.7%	6	4.1%	0.9–7.3%
*Cryptosporidium* ^#^	0	—	—	3	5.7%	0–12.1%^#^	3	4%	0–8.4%
*Porrocaecum*	0	—	—	4	4.8%	0.2–9.4%	4	2.7%	0.1–5.4%
*Porrocaecum*-Capillarids	0	—	—	3	3.6%	0–7.6%	3	2%	0–4.4%
*Syngamus*-Capillarids	1	1.6%	0–4.7%	0	—	—	1	0.7%	0–2%

Total	17	27%	16–37.9%	35	42.2%	31.5–62.8%	52	35.6%	27.8–43.4%

*Assemblage

^#^Values for *G. duodenalis *and *Cryptosporidium* were calculated based on analysis of samples from 23 pet birds, 52 zoo birds, and a total number of 75 birds.

**Table 4:** Clinical signs and results of coprological examination in symptomatic pet and zoo birds (*n* = 10).

Birds	Origin	Clinical signs	Results of coprological examination
Pea fowl	Pet bird	Anorexia, depression, ruffled feathers	*Syngamus*-Capillarids
Grey Crowned Crane	Zoo bird	Anorexia, diarrhea, ruffled feathers, skeletal abnormalities, stunted growth, weakness	*Porrocaecum-*Capillarids
Grey Crowned Crane	Zoo bird	Anorexia, diarrhea, ruffled feathers, skeletal abnormalities, stunted growth, weakness	*Porrocaecum-*Capillarids
Canary	Pet bird	Diarrhoea, depression	Coccidia
Canary	Pet bird	Anorexia, depression, ruffled feathers	Coccidia
Canary	Pet bird	Anorexia, depression	Coccidia
Blue-fronted Amazon	Pet bird	Anorexia, depression	Negative
Fischer's Lovebird	Pet bird	Depression	Negative
Fischer's Lovebird	Pet bird	Diarrhoea, ruffled feathers	Negative
Budgerigar	Pet bird	Diarrhoea	Coccidia
